# Clinical outcomes of DMEK comparing endothelium-out injector and endothelium-in pull-through techniques in Asian eyes

**DOI:** 10.3389/fmed.2025.1555620

**Published:** 2025-04-03

**Authors:** Ezekiel Ze Ken Cheong, Clarissa Ng Yin Ling, Qiu Ying Wong, Chloe Si Qi Chua, Hla Myint Htoon, Marcus Ang

**Affiliations:** ^1^Ophthalmology and Visual Sciences Academic Clinical Program, Duke-NUS Medical School, Singapore, Singapore; ^2^Singapore National Eye Centre, Singapore, Singapore; ^3^Singapore Eye Research Institute, Singapore, Singapore

**Keywords:** Descemet membrane endothelial keratoplasty (DMEK), clinical outcomes, graft survival, surgical techniques, pull-through, endothelium-in

## Abstract

This is a prospective interventional study of 180 consecutive Descemet membrane endothelial keratoplasty (DMEK) cases, comparing injector (endothelium-out) and pull-through (endothelium-in) surgical techniques in Asian eyes. The main outcome measures were 5-year graft survival and intraoperative and postoperative complications. In our study, a pull-through technique for DMEK was employed more frequently in PBK (66.2%) than in FECD (10.7%) eyes (*p* < 0.001). Overall 5-year graft survival was 90% (98% in FECD and 64% in PBK eyes; *p* < 0.001). We observed higher rates of intraoperative donor graft tears (6.5% vs. 0.8%; *p* = 0.049) and persistent postoperative corneal edema (19.4% vs. 6.8%; *p* = 0.022) in pull-through DMEK than injector DMEK. However, multivariable analysis suggested that surgical technique was not a significant factor associated with graft survival, that is, PBK as the surgical indication was the main factor associated with graft failure (hazard ratio = 12.5; *p* < 0.01) and postoperative complications (odds ratio = 4.41; *p* < 0.01), regardless of surgical technique used. In our Asian study cohort, both injector (endothelium-out) and pull-through (endothelium-in) surgical techniques for DMEK had comparable clinical outcomes, when adjusted for confounders.

## Introduction

Descemet membrane endothelial keratoplasty (DMEK) is gaining popularity (1) as a treatment for diseased or damaged Descemet membranes and corneal endothelium (2). Compared to Descemet stripping automated endothelial keratoplasty (DSAEK), DMEK has the potential for faster visual recovery (3) and lower complication and rejection rates (4), despite being technically more challenging (5). Despite these advantages, complication and failure rates remain higher for advanced pseudophakic bullous keratopathy (PBK), compared with eyes with Fuchs endothelial cell dystrophy (FECD) (6, 7). As such, DMEK techniques have been continually refined for efficiency and effectiveness (8), especially for PBK patients with poorer prognoses.

**Figure 1:**
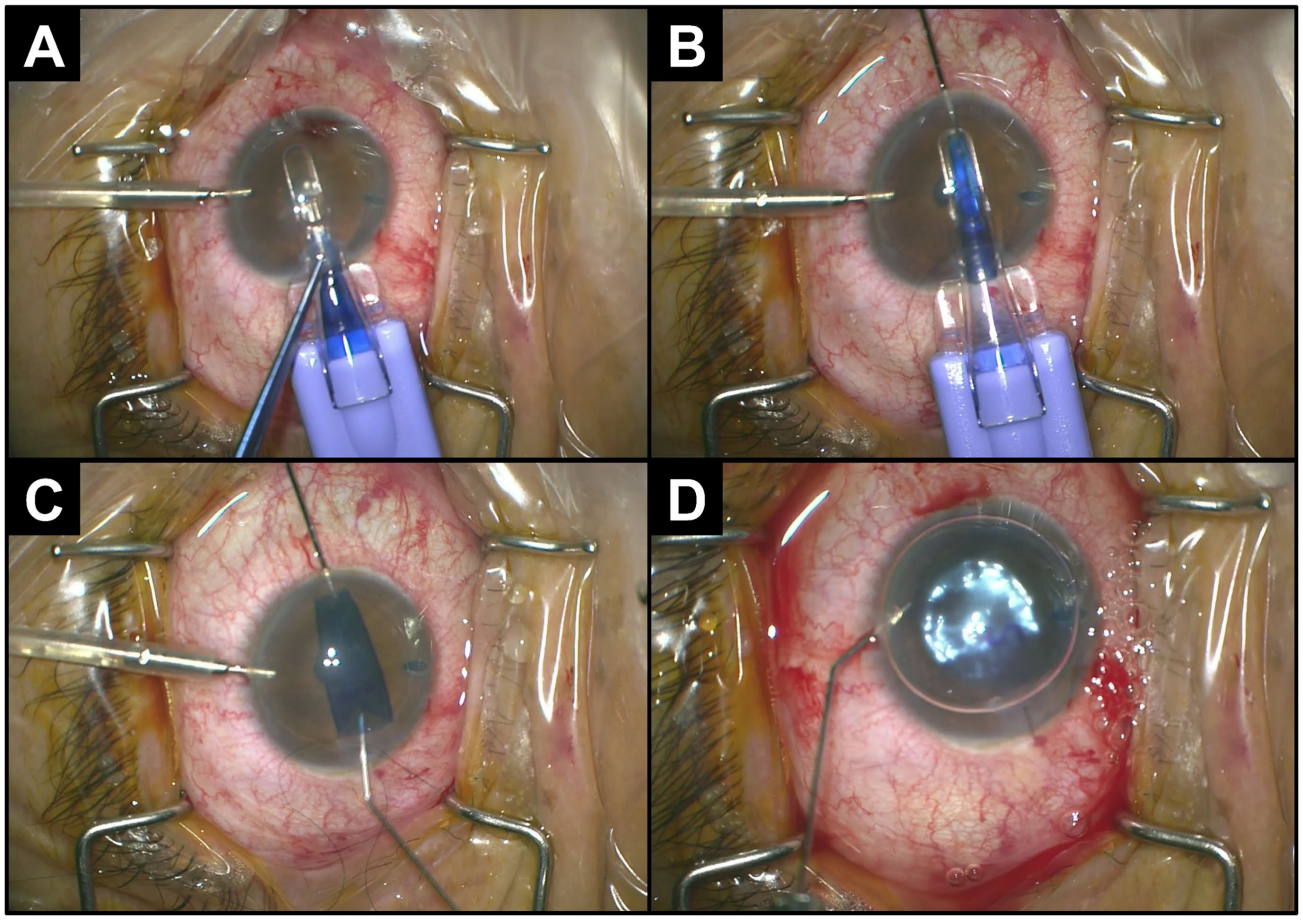
Intraoperative procedure for pull-through DMEK. **(A)** Insertion of the cartridge into the anterior chamber through a clear cornea incision. **(B)** Grasping and pulling-through of graft with forceps. **(C)** Natural unfolding of the graft with endothelium-down. **(D)** Injection of gas to tamponade the donor graft to the recipient cornea.

Recently, DMEK donor insertion with the endothelium folded inward (“endothelium-in”) and “pulled-through” into the anterior chamber has been described (Figure 1) (9–11). This has some advantages over the traditional injector technique as DMEK donors naturally scroll up with the endothelial layer facing outward (“endothelium-out”) (12, 13). Ensuring the graft adopts the correct orientation (“endothelium-down”) after unscrolling requires surgical dexterity and maneuvers, which can possibly result in intraoperative endothelial cell loss, especially for surgeons with less experience (14, 15). Pull-through techniques have been reported to have quicker unfolding and positioning times than injector techniques (16, 17). This may lead to reduced surgical manipulation (18) and less endothelial cell exposure to shearing forces (19). Pull-through techniques may thus provide surgical predictability and control for challenging eyes such as those with a previous vitrectomy, fixated intraocular lens (IOL), or iris abnormalities (20).

Current studies for pull-through DMEK report reduced intraoperative endothelial cell losses (11, 21), with similar clinical outcomes to injector techniques (19, 22). However, there is currently a lack of direct comparative studies assessing clinical outcomes between pull-through and injector DMEK techniques, especially in the Asian context where surgical indications and challenges might differ (23, 24). Thus, we present here our report on the outcomes of consecutive DMEKs completed with both injector and pull-through techniques in our local population of Asian eyes—with a focus on graft survival and intraoperative and postoperative complications.

## Methods

### Study design and participants

We conducted a prospective interventional study of 180 consecutive DMEK surgeries completed by a single cornea specialist (MA) at a tertiary ophthalmology center—Singapore National Eye Centre—from July 2016 to September 2023. All DMEKs in that timeframe completed by the cornea surgeon for the indications of FECD and PBK were included. DMEK as a regraft for failed previous keratoplasties was excluded to reduce confounding ocular comorbidities. Our study was conducted as part of the Singapore Corneal Transplant Registry, which monitors clinical data and outcomes of corneal transplants in Singapore (25), with ethics approval from the local institutional review board (CIRB Ref 2011/577/A) and in accordance with the Declaration of Helsinki. Written informed consent was obtained from all subjects. All cases were at various stages of follow-up. Basic demographic data, clinical outcomes, best-corrected visual acuity (BCVA), ECD, and DMEK donor graft details were compiled from electronic health records.

### Surgical procedure

All donor tissues were obtained from the Singapore Eye Bank and underwent stringent monitoring and quality assurance. For the intraoperative procedure, techniques previously described were used (10, 26, 27). All grafts were prepared intraoperatively by the surgeon and were not preloaded. Essentially, the donor cornea was prepared by the surgeon using the SCUBA technique (28). This was followed by donor graft trephination according to size. In this series, the employment of an injector or pull-through technique was based on the surgeon’s choice. In general, pull-through DMEK was chosen for eyes with greater surgical challenges such as PBK eyes with poor anterior chamber visualization. For the standard injector technique, the stained DMEK graft was inserted using a glass injector (Geuder AG, Germany) while in the natural endothelium-out scroll conformation and unfolded into the endothelium-down orientation using air and a balanced salt solution in a shallow anterior chamber as previously described (26, 29). For the pull-through technique, the DMEK graft was tri-folded after staining, pulled into the EndoGlide cartridge (CORONET DMEK EndoGlide; Network Medical Products, United Kingdom), and pulled through into the anterior chamber in the endothelium-down orientation (Figure 1); 20% sulfur hexafluoride gas was injected to achieve 80% fill to tamponade the graft. All wounds, if required, were closed with a single 10/0 nylon suture.

### Postoperative management

As previously described (27), all patients remained in a face-up posture for at least 2 h and had IOP routinely assessed and managed with topical treatment before discharge. All patients received standard postoperative topical antibiotics (levofloxacin 0.5%; Santen, Japan) and topical corticosteroid regime (prednisolone acetate; Allergan, United States) following a standard tapering dose as previously described (30). Patients generally had follow-up visits at 1 day, 1 week, 1 month, 3 months, and 6 months postoperatively, after which they were continually followed up every 6 months. Given that pull-through DMEK is a relatively newer technique than the more established and traditional injector technique, most of the pull-through cases did not yet have visits beyond the 24-month mark. At each follow-up visit, they were examined by slit-lamp biomicroscopy, and BCVA was measured using the Snellen chart and subsequently converted to logarithm of the minimum angle of resolution (logMAR) units for statistical analysis (31). ECD measurements were obtained via non-contact specular microscopy (CellChek 20; Konan Medical Corp, Japan) by certified ophthalmic technicians as previously described (32). ECD measurements were obtained from the built-in automatic endothelial cell segmentation software, using the center method to measure cell area. ECD measurement was not part of routine testing at every follow-up and was only ordered for patients requiring closer monitoring or non-improving vision.

### Clinical outcomes

Intraoperative complications recorded were as previously defined (27): DMEK donor graft tears, decentered graft placements, development of any bleeding (hyphema), high vitreous pressure, and aqueous misdirection defined as a flat anterior chamber with high intraocular pressure (IOP) in the presence of a patent peripheral iridectomy. Postoperative complications recorded include the following: cystoid macular edema, persistent cornea edema or haze (without detachment or rejection), early signs of graft rejection (keratic precipitates, stromal infiltrates, and Khodadoust lines), partial and complete detachments (lack of adherence of <30% and ≥30%, respectively, of the graft surface area) (30), need for rebubbling, retinal detachment, and new-onset glaucoma or ocular hypertension. Graft failure was defined as persistent, irreversible loss of corneal clarity irrespective of visual acuity, from any cause (33). Surviving grafts were defined as clear and functional grafts without an outcome of graft failure.

### Statistical analyses

SPSS 26.0 (SPSS; IBM Corp, United States) and GraphPad Prism (Prism; GraphPad, United States) were used for all statistical analyses in this study. Descriptive statistics included mean ± standard deviation for continuous variables, whereas categorical variables included frequency distribution and percentages in parentheses. All between-group comparisons of continuous parameters were performed using independent *t*-tests (paired for applicable comparisons). All between-group comparisons of categorical parameters were performed using Fisher’s exact or chi-square tests. Graft survival was analyzed using Kaplan–Meier plots, log-rank tests, and Cox regression analysis. Multivariable analysis models were built using potentially confounding independent variables that were found to be significantly different in the descriptive characteristics of the cohort. Cox regression analysis of graft survival was reported as hazard ratio (HR), logistic regression analyses of intraoperative and postoperative complications were reported as odds ratio (OR), and linear regression of endothelial cell loss (ECL) was reported as beta coefficient. Upper and lower 95% confidence intervals (CIs) were labeled in graphs and as a range for HR, OR, and beta. Tests were all two-sided if applicable, with statistical significance set at a *p*-value of <0.05. Statistical significance was indicated with a single asterisk for *p* < 0.05, a double asterisk for *p* < 0.01, and a triple asterisk for *p* < 0.001. ECD and BCVA were analyzed for up to 24 months post-DMEK. ECL was defined as the percentage loss of ECD compared to the preoperative donor ECD.

## Results

We analyzed 180 eyes of 160 subjects who underwent DMEK at a mean age of 69.5 ± 10.1 years (Table 1). In total, 65.6% of eyes underwent injector DMEK (*n* = 118), and 34.4% of eyes underwent pull-through DMEK (*n* = 62); 31.4% (*n* = 37) of the injector-DMEK cases and 62.9% (*n* = 39) of the pull-through DMEK cases were male (*p* < 0.001); 57.2% of surgical indications were FECD (*n* = 103) and 42.8% were PBK (*n* = 77). Pull-through DMEK was mostly employed for surgically challenging eyes; 82.3% (*n* = 51) were PBK eyes, whereas only 22.0% (*n* = 26) of injector DMEKs were PBK eyes (*p* < 0.001). Pull-through DMEK was also associated with a greater rate of prior glaucoma before surgery (*p* < 0.001).

**Table 1:** Descriptive characteristics of 180 DMEK recipient eyes and donor DMEK grafts.

Characteristics	All eyes *n* = 180	Insertion technique		*P*
		Injector	Pull-through	
		*n* = 118	*n* = 62	
Recipient characteristics				
Age at DMEK	69.5 ± 10.1	69.9 ± 9.1	68.8 ± 11.8	0.493
Ethnicity				
Chinese	155 (86.1)	105 (89.0)	50 (80.6)	0.472
Indian	6 (3.3)	3 (2.5)	3 (4.8)	
Malay	6 (3.3)	3 (2.5)	3 (4.8)	
Others	13 (7.2)	7 (5.9)	6 (9.7)	
Sex				
Male	76 (42.2)	37 (31.4)	39 (62.9)	<0.001***
Female	104 (57.8)	81 (68.6)	23 (37.1)	
Glaucoma pre-DMEK				
Glaucomatous	45 (25.0)	19 (16.1)	26 (41.9)	<0.001***
Non-glaucomatous	135 (75.0)	99 (83.9)	36 (58.1)	
Indication for DMEK				
FECD	103 (57.2)	92 (78.0)	11 (17.7)	<0.001***
PBK	77 (42.8)	26 (22.0)	51 (82.3)	
Donor characteristics				
Age*	62.4 ± 7.5	62.0 ± 7.4	63.9 ± 7.7	0.397
Sex*				
Male	111 (64.2)	74 (64.9)	37 (62.7)	0.297
Female	62 (35.8)	30 (35.1)	22 (37.3)	
ECD	2,833 ± 223	2,838 ± 211	2,825 ± 246	0.709
CV†	34.1 ± 3.7	34.7 ± 3.8	33.1 ± 3.4	<0.01**
HEX†	57.4 ± 6.9	56.3 ± 7.0	59.4 ± 6.3	<0.01**

FECD, Fuchs endothelial cell dystrophy; PBK, pseudophakic bullous keratopathy; BCVA, best-corrected visual acuity; CV, coefficient of variation; HEX, hexagonality.

*Donor age and gender data: 114 injector, 59 pull-through, 173 total.

†Donor CV and HEX data: 111 injector, 61 pull-through, 172 total.

### Clinical outcomes of injector vs. pull-through techniques

The clinical outcomes of both injector and pull-through techniques, in terms of complications and final graft outcomes, are compared in Table 2. We observed a higher rate of intraoperative donor graft tear (6.5% vs. 0.8%; *p* = 0.049) and postoperative corneal edema (19.4% vs. 6.8%; *p* = 0.022) in pull-through DMEK than injector DMEK. We also noted early signs of immune graft rejection in 4.4% (*n* = 8) of eyes; however, all were single episodes that fully resolved with increased topical steroids, and all grafts recovered clarity and remained clear. Of the 16 eyes (8.9%) with partial or complete graft detachment, eight required rebubbling to reattach the graft (4.4% of overall rebubbling rate). All rebubbling procedures were successful. The choice of surgical technique, injector or pull-through, was not found to be associated with different outcomes of graft survival (*p* = 0.192). Subgroup analysis within the two DMEK indications of FECD (Supplementary Table 1) and PBK (Supplementary Table 2) was also used to compare the clinical outcomes of the two techniques. There was no significant difference detected between the injector and pull-through techniques in both the FECD and PBK subgroups. Within the PBK cases, pull-through DMEK did not show significantly greater rates of intraoperative graft tears (7.8% vs. 0%; *p* = 0.294) or postoperative cornea edema (23.5% vs. 15.4%; *p* = 0.556).

**Table 2:** Comparison of visual outcomes, intraoperative, postoperative complications, and final graft outcomes between injector and pull-through surgical techniques.

Outcomes	All eyes *n* = 180	Insertion technique		*P*
		Injector	Pull-through	
		*n* = 118	*n* = 62	
Intraoperative complications				
Any complication	21 (11.7)	10 (8.5)	11 (17.7)	0.087
Donor graft tear	5 (2.8)	1 (0.8)	4 (6.5)	0.049*
Aqueous misdirection	3 (1.7)	2 (1.7)	1 (1.6)	1.00
Hyphema	8 (4.4)	5 (4.2)	3 (4.8)	1.00
High vitreous pressure	2 (1.1)	1 (0.8)	1 (1.6)	1.00
Decentered graft	5 (2.8)	2 (1.7)	3 (4.8)	0.341
Postoperative complications				
Any complication	48 (26.7)	26 (22.0)	22 (35.5)	0.075
Cystoid macula edema	7 (3.9)	6 (5.1)	1 (1.6)	0.425
Early rejection signs	8 (4.4)	5 (4.2)	3 (4.8)	1.00
Partial detachment	13 (7.2)	9 (7.6)	4 (6.5)	1.00
Complete detachment	3 (1.7)	1 (0.8)	2 (3.2)	0.273
Rebubbling required	8 (4.4)	5 (4.2)	3 (4.8)	1.00
Corneal haze/edema	20 (11.1)	8 (6.8)	12 (19.4)	0.022*
Ocular hypertension	9 (5.0)	5 (4.2)	4 (6.5)	0.497
Retinal detachment	2 (1.1)	1 (0.8)	1 (1.6)	1.00
Final graft outcome				
Clear and surviving	169 (93.8)	113 (95.8)	56 (90.3)	0.192
Graft failure	11 (6.1)	5 (4.2)	6 (9.7)	
Visual outcomes (logMAR)				
Pre-DMEK BCVA	0.96 ± 0.61	0.76 ± 0.45	1.35 ± 0.67	<0.001***
Post-DMEK BCVA*	0.30 ± 0.37	0.24 ± 0.31	0.41 ± 0.43	<0.01**
Post-DMEK BCVA ≥6/12	132 (73.3)	94 (79.7)	38 (61.3)	0.013*
% improvement in BCVA	66.4 ± 35.3	66.0 ± 37.8	67.2 ± 30.2	0.828

logMAR, logarithm of the minimum angle of resolution; BCVA, best-corrected visual acuity. *Postop BCVA defined as the best BCVA score within 24 months postoperatively.

### DMEK graft survival

**Figure 2:**
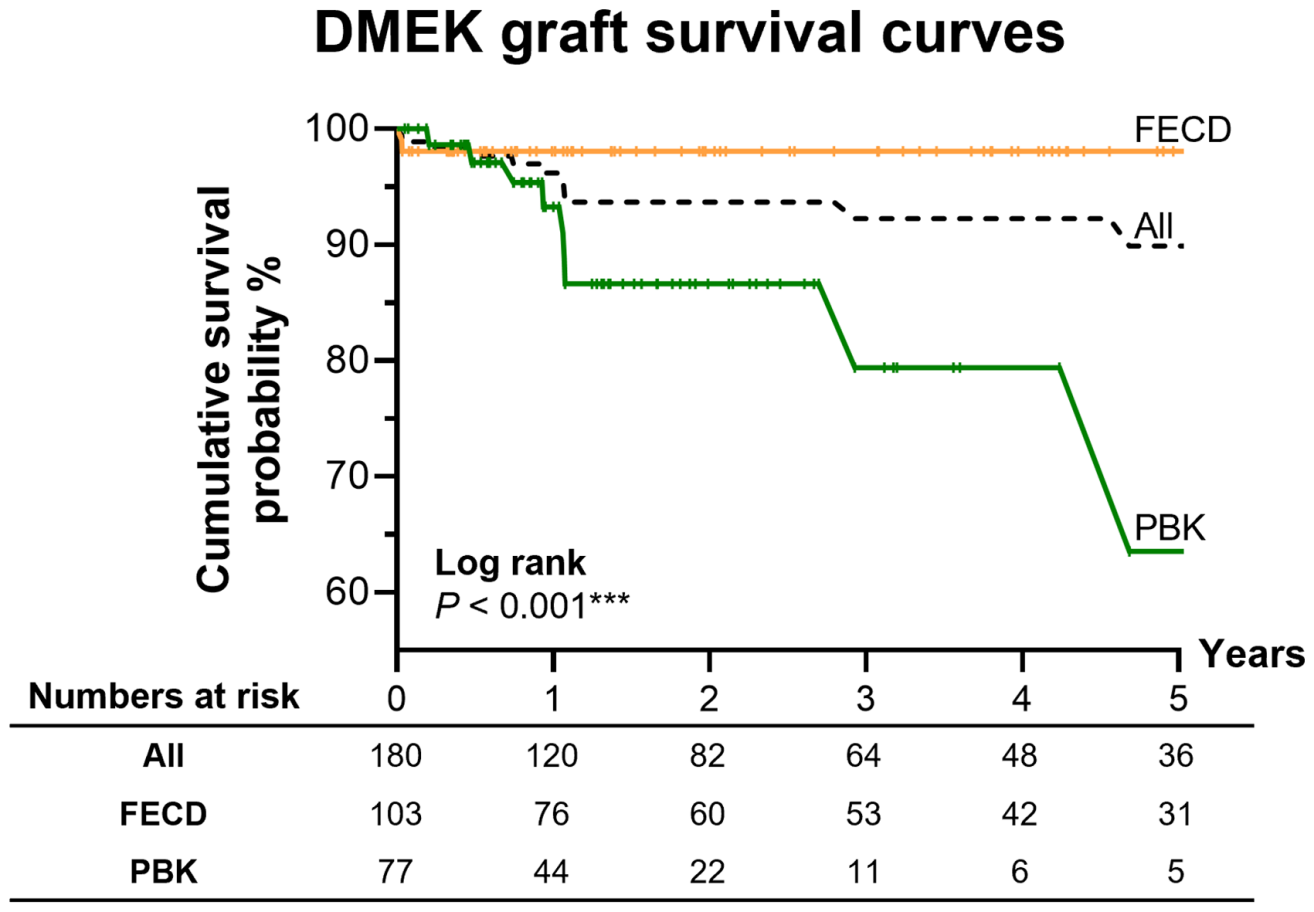
Kaplan–Meier curve of cumulative graft survival probability. DMEK graft survival curves over 5 years, comparing FECD and PBK. Overall 5-year survival was 90% (95% CIs = [86–94%]). The numbers at risk at each year across 5 years are indicated in the table below each graph. Survival curves were significantly different when comparing indications (log-rank *p* < 0.001) of FECD and PBK.

The 5-year overall cumulative graft survival (Figure 2) was 90% (95% CIs = [86–94%]), significantly greater for the FECD group than the PBK group (98% vs. 64%; log-rank *p* < 0.001). Survival curves for the two different techniques (injector vs. pull-through) were significantly different (log-rank *p* = 0.017). However, comparing within the FECD and PBK groups, there were no significant differences in the survival curves between the pull-through and injector techniques (log-rank *p* = 0.624 and 0.258, respectively). Cox regression analysis (Table 3) suggested that the pull-through technique was associated with graft failure in the univariable analysis (HR = 10.5; 95% CIs = [2.42–55.9]; *p* < 0.01). However, after adjusting for age, sex, prior glaucoma, and surgical indication in the multivariable analysis, the association was no longer statistically significant (HR = 2.64; 95% CIs = [0.526–16.3]; *p* = 0.256). However, PBK remained significantly associated with graft failure (HR = 12.5; 95% CIs = [2.11–101]; *p* < 0.01) when compared to FECD.

**Table 3:** Cox regression of DMEK graft survival using multiple variables.

Variables	A: Univariable		B: Multivariable	
	Hazard ratio (95% CIs)	*P*	Hazard ratio (95% CIs)	*P*
Age at DMEK	0.978 (0.915–1.04)	0.491	0.961 (0.899–1.03)	0.236
Male sex	2.34 (0.698–8.19)	0.164	0.877 (0.208−3.63)	0.854
Prior glaucoma	1.57 (0.340–5.58)	0.509	0.752 (0.154–2.96)	0.696
DMEK indication of PBK (vs. FECD)	13.5 (3.29–91.1)	<0.01**	12.5 (2.11–101)	<0.01**
Pull-through technique (vs. injector)	10.5 (2.42–55.9)	<0.01**	2.64 (0.526–16.3)	0.256

PBK, pseudophakic bullous keratopathy; FECD, Fuchs endothelial cell dystrophy.

### Multivariable analysis of intraoperative and postoperative complications

We observed that the pull-through technique was associated with higher rates of intraoperative donor graft tear and postoperative corneal edema (Table 2). However, given that there were many possible confounders as shown in Table 1, a multivariable analysis was conducted. We observed that the pull-through technique, after adjusting for age, sex, prior glaucoma, and DMEK indication, was not associated with increased incidence of any intraoperative (OR = 1.38; 95% CIs = [0.436–4.38]; *p* = 0.581) or postoperative complications (OR = 0.93; 95% CIs = [0.384–2.19]; *p* = 0.871). Specifically, it was not significantly associated with intraoperative graft tear (OR = 5.18; 95% CIs = [0.425–136]; *p* = 0.232) nor persistent postoperative corneal edema (OR = 1.51; 95% CIs = [0.467–5.15]; *p* = 0.494). However, we found that PBK remained significantly associated with postoperative corneal edema (OR = 7.69; 95% CIs = [1.83–40.7]; *p* < 0.01) and other postoperative complications (OR = 4.41; 95% CIs = [1.78–11.3]; *p* < 0.01), unlike FECD.

### Postoperative endothelial cell density and loss

**Figure 3:**
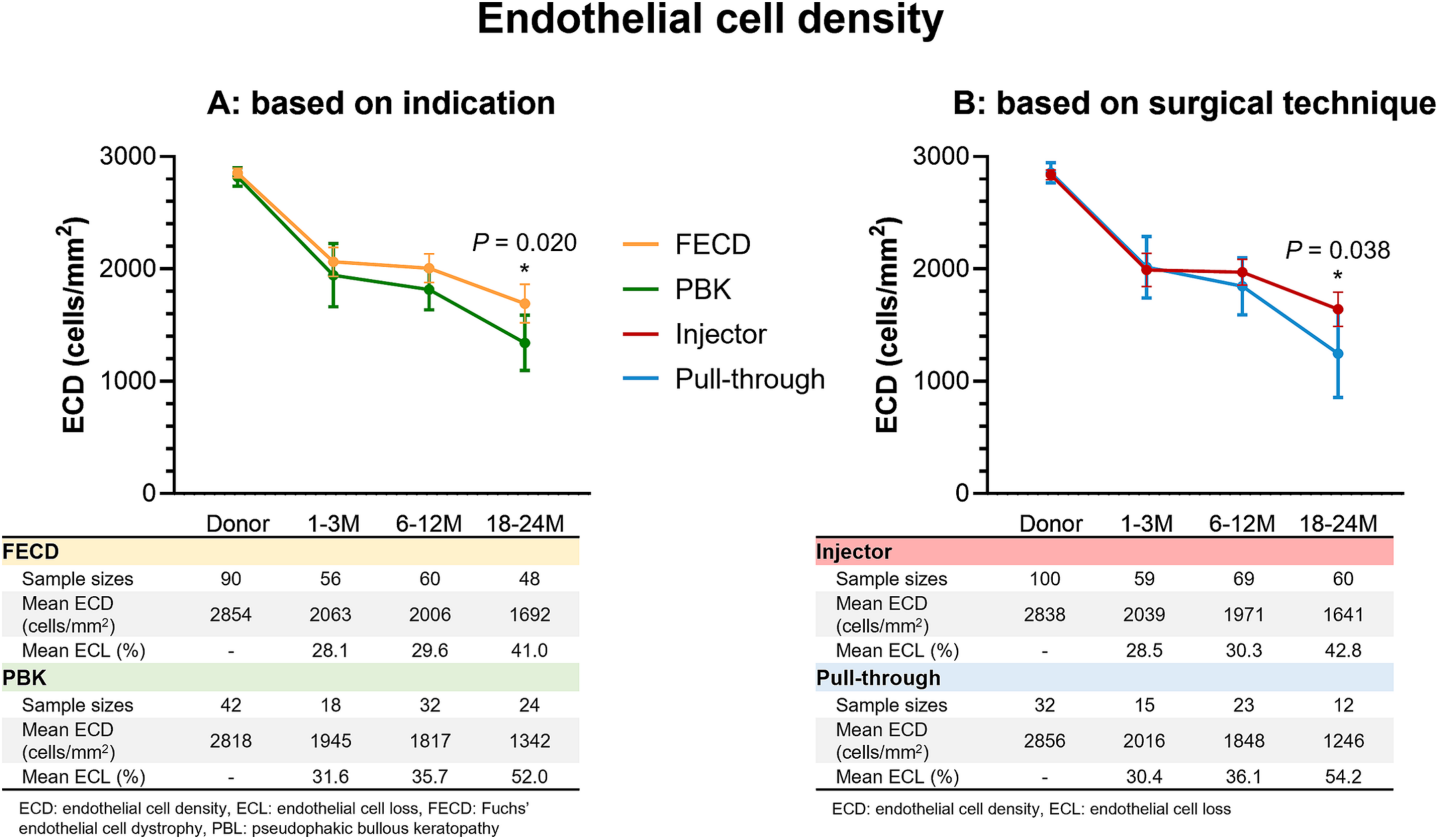
Post-DMEK endothelial cell density and loss. Post-DMEK endothelial cell density (ECD) and endothelial cell loss (ECL) at 1–3 months, 6–12 months, and 18–24 months post-DMEK, comparing **(A)** FECD (yellow) and PBK (green) DMEK indications and **(B)** injector (red) and pull-through (blue) surgical techniques. 95% of CIs are indicated as vertical error bars at each timepoint. ECD was significantly different at 18–24 months post-DMEK for comparisons between surgical indications (*p* = 0.020) and surgical techniques (*p* = 0.038).

The mean donor ECD was 2,833 ± 223 cells/mm^2^. Figure 3 shows the ECD at various timepoints post-DMEK for the different surgical indications and techniques utilized. Mean ECD was not significantly different between injector and pull-through DMEK within the first 3 months (2,039 vs. 2,016 cells/mm^2^; *p* = 0.875) or within 6 to 12 months (1,941 vs. 1,848 cells/mm^2^; *p* = 0.311). Significant differences were detected from 18 to 24 months when comparing between FECD and PBK (1,692 vs. 1,342 cells/mm^2^; *p* = 0.020) and between injector and pull-through techniques (1,637 vs. 1,246 cells/mm^2^; *p* = 0.038). However, there were no differences when comparing the ECL between injector and pull-through at all timepoints: 1 to 3 months (28.5% vs. 30.4%; *p* = 0.709), 6 to 12 months (30.3% vs. 36.1%; *p* = 0.166), and 18 to 24 months (54.2% vs. 42.8%; *p* = 0.083). The multivariable analysis did not find any variable that was associated with significantly different ECLs at all three timepoints.

### Visual outcomes

Preoperative and postoperative BCVA are described in Table 2. Eyes undergoing pull-through DMEK had poorer BCVA preoperatively (logMAR 1.35 vs. 0.76; *p* < 0.001) and postoperatively (logMAR 0.41 vs. 0.24; *p* < 0.01) but did not have any significant different percentage improvement in BCVA post-DMEK compared to injector DMEK (67.2% vs. 66.0%; *p* = 0.828). Overall, 79.7% (*n* = 94) of injector-DMEKs and 61.3% (*n* = 38) of pull-through DMEKs achieved a postoperative BCVA of 6/12 or better (*p* = 0.013). Longitudinally, FECD eyes had better visual outcomes than PBK: preoperatively (logMAR 0.68 vs. 1.33; *p* < 0.001), 1 to 3 months post-DMEK (logMAR 0.32 vs. 0.43; *p* = 0.047), 6 to 12 months post-DMEK (logMAR 0.25 vs. 0.43; *p* < 0.01), and 18 to 24 months post-DMEK (logMAR 0.25 vs. 0.61; *p* < 0.001).

## Discussion

In our prospective study of consecutive DMEK cases performed in Asian eyes with 57.2% FECD, the overall 5-year graft survival was 90% (98% in FECD, 64% in PBK), which is similar to the findings of Price et al. (34), who showed 93% in FECD eyes, and Birbal et al. (35), who showed 90% in a cohort of 89.2% FECD eyes. In our cases, PBK was significantly associated with graft failure (HR = 12.5; *p* < 0.01) and incidence of postoperative complications (OR = 4.41; *p* < 0.01). The pull-through donor insertion technique had comparable graft survival compared to the injector insertion technique when adjusted for the indication of PBK, agreeing with the recent systematic review of Ong et al. (11) and the findings of Price et al. (19).

We did observe an overall higher rate of intraoperative graft tears in pull-through DMEK (6.5% vs. 0.8%; *p* = 0.049). There is overall a risk of small peripheral tears due to the pulling-through of the graft with forceps (9), as opposed to the injector technique which does not involve grasping during the graft insertion phase of DMEK. We, however, did not note any tears or significant difficulties during the loading and preparation of the grafts in either method. There was a higher rate of persistent postoperative corneal edema in our pull-through cases (19.4% vs. 6.8%; *p* = 0.022). However, our multivariable analysis adjusted for confounders, suggesting that PBK is the main contributing factor (OR = 7.69; *p* < 0.01), rather than the pull-through technique (OR = 1.51; *p* = 0.494). We did not observe any difference in rebubbling rate for pull-through vs. injector techniques (4.8% vs. 4.2%; *p* > 0.99), which agrees with the findings of other comparative studies (11, 19, 36).

We found no significant difference in ECL between the injector and pull-through DMEK in the postoperative period up to 24 months. The multivariable analysis did not highlight pull-through as a significant variable for increased ECL. The 6- to 12-month post-DMEK ECL in our pull-through cases was 30.3%, compared to another study showing a pooled ECL of 28.1% at 6 months and 29.6% at 12 months (11). In terms of visual outcomes, we found that eyes with FECD had better 2-year postoperative BCVA than PBK eyes, at all timepoints, which is consistent with previous reports(37, 38). Nonetheless, we found that the percentage improvement in BCVA after surgery was similar when comparing both surgical indications and surgical techniques. Overall, our findings suggest that PBK was the most important independent factor for graft failure, postoperative complications, and poorer visual outcomes, all of which are consistent with the current literature (6, 8).

The potential advantages of the pull-through technique include the minimization of endothelial contact with the insertion device (22) and quicker graft unfolding and positioning times due to the spontaneous unfurling into its natural endothelium-out configuration (39). The pull-through technique could also be useful in younger donor grafts, given their tendency to form tighter scrolls than older donors (40, 41). In addition, the pull-through insertion method results in better control of the donor graft (18), whereas the graft is left free-floating in the anterior chamber in the injection method (42). This element of graft handling is especially crucial in eyes with difficult visualization and shallow anterior chambers (43), which are more common in Asian eyes due to darker irises and narrower palpebral fissures with smaller, deeper-set eyes (23). In addition, pulling-through also circumvents reliance on normal anterior segment structures such as an intact iris diaphragm (44), increasing its utility for eyes with aniridia or iridocorneal syndrome (45). Potential concerns of holding the graft with forceps in pull-through DMEK are peripheral graft tears and endothelial cell damage with intraoperative ECL. Forceps-free endothelium-in injection of the DMEK graft has been described (46), which may help reduce the graft tears or ECL, but this removes the benefit of bimanual graft control afforded by the pull-through technique. In addition, the endothelial cell area potentially damaged by each forceps bite is relatively minimal and confined to only one distal end of the graft periphery (9). A novel infusion forceps for pull-through DMEK has been developed, which can grasp the donor tissue and additionally control anterior chamber depth at the same time (47), highlighting the continual innovation for DMEK techniques and devices.

Key strengths of our study are that we prospectively analyzed consecutive DMEK outcomes which minimizes selection bias and represents real-world results of the two surgical techniques. We also reported a relatively large sample size and analyzed a broad range of clinical outcomes such as graft survival, complications, ECL, and visual outcomes. One limitation of our study is that pull-through DMEK was more likely to be chosen for surgically challenging eyes (PBK, prior glaucoma, etc.), with a likely poorer prognosis. However, we mitigated this by adjusting for confounders in our multivariable analyses and reported no significant difference in outcomes between pull-through and injector DMEK, when performed by an experienced cornea surgeon. Furthermore, it would not have been feasible to randomize surgical techniques in our patient population. Nonetheless, more comprehensive multicenter studies and/or randomized controlled trials are required to conclusively compare injector and pull-through techniques (11), especially given the relative novelty of pull-through DMEK.

We recognize that DMEK indications, surgical complexity, and clinical practice in the global population might differ from our experience but, nonetheless, we strived to contribute to the growing body of knowledge for pull-through DMEKs and to provide valuable insight into DMEK techniques and outcomes in our local Asian population. In our experience, pull-through DMEK for complex eyes has been a valuable technique, with the controlled intraoperative graft unfolding helping alleviate potential hurdles such as an abnormal anterior segment anatomy or fixated IOLs. We demonstrated that pulling-through could be a good choice for PBK patients, especially those who might have poor corneal clarity for intraoperative visualization, given the similar clinical outcomes in pull-through and injector DMEKs for PBK. This prospective DMEK study had varying follow-up periods and sample sizes; nonetheless, our routine and serial follow-up compared favorably with other DMEK studies (37, 38). Finally, we would have ideally also measured and compared graft preparation, unfolding, and total operation times to be able to report a comprehensive comparison of the surgical techniques.

To conclude, our comparative study on consecutive DMEK suggests that the pull-through technique could have comparable clinical outcomes with traditional donor injector techniques, despite being employed for the most challenging cases. This was demonstrated in multivariate analyses with adjustment for confounders such as prior glaucoma and PBK as a surgical indication. Given the challenges of DMEK graft insertion and positioning for some eyes, pull-through DMEK may be a useful technique that offers better graft control.

## Data Availability

The original contributions presented in the study are included in the article/Supplementary material, further inquiries can be directed to the corresponding author.
