# Corrigendum: Clinical outcomes of DMEK comparing endothelium-out injector and endothelium-in pull-through techniques in Asian eyes

**DOI:** 10.3389/fmed.2025.1624456

**Published:** 2025-06-24

**Authors:** Ezekiel Ze Ken Cheong, Clarissa Ng Yin Ling, Qiu Ying Wong, Chloe Si Qi Chua, Hla Myint Htoon, Marcus Ang

**Affiliations:** ^1^Ophthalmology and Visual Sciences Academic Clinical Program, Duke-NUS Medical School, Singapore, Singapore; ^2^Singapore National Eye Centre, Singapore, Singapore; ^3^Singapore Eye Research Institute, Singapore, Singapore

**Keywords:** Descemet membrane endothelial keratoplasty (DMEK), clinical outcomes, graft survival, surgical techniques, pull-through, endothelium-in

In the published article, there was an error in Figure 1 as published. Panels C and D of Figure 1 have been erroneously swapped. The corrected Figure 1 with its unchanged caption appear below.

**Figure 1:**
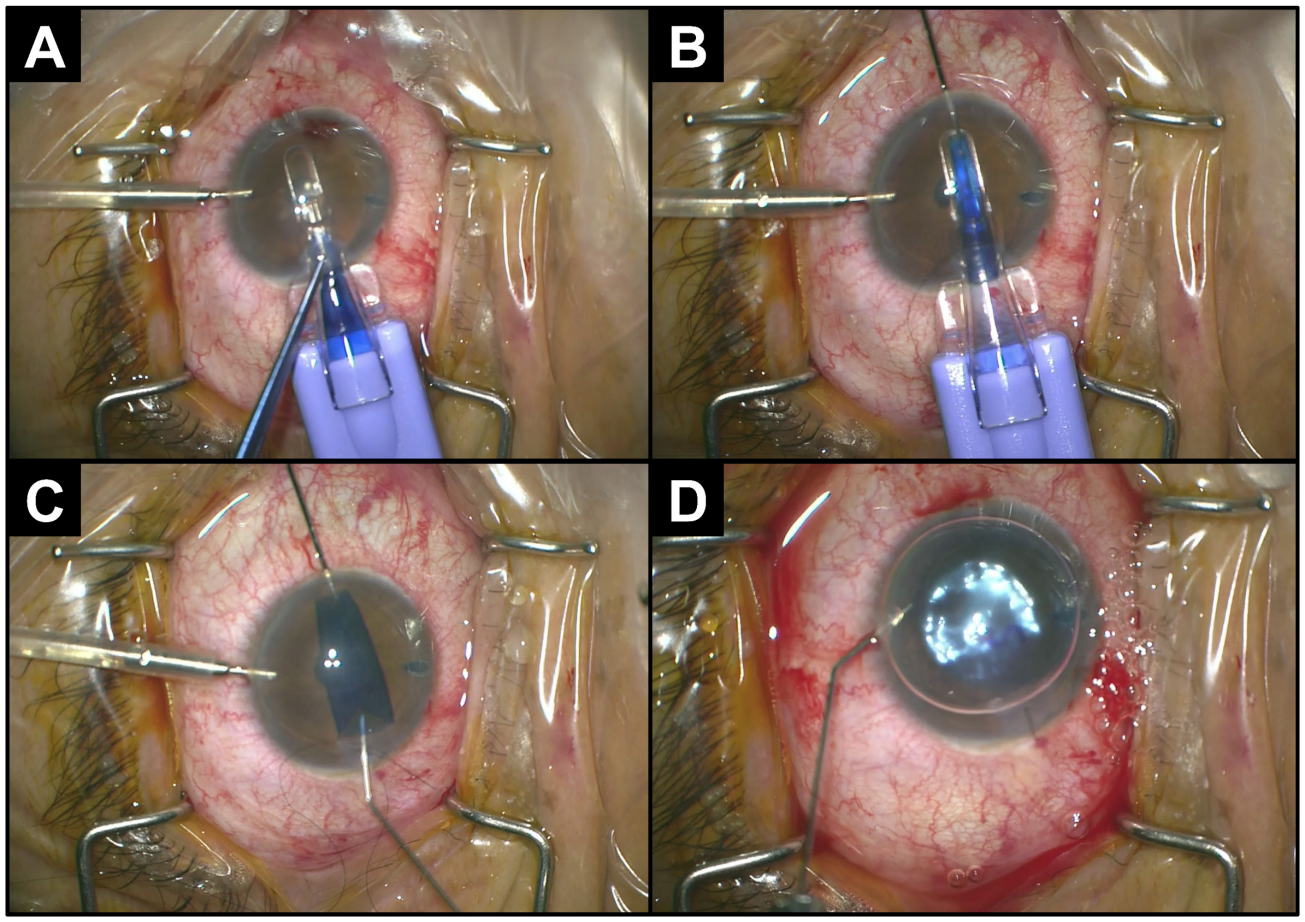
Intraoperative procedure for pull-through DMEK. **(A)** Insertion of the cartridge into the anterior chamber through a clear cornea incision. **(B)** Grasping and pulling-through of graft with forceps. **(C)** Natural unfolding of the graft with endothelium-down. **(D)** Injection of gas to tamponade the donor graft to the recipient cornea.

The authors apologize for this error and state that this does not change the scientific conclusions of the article in any way. The original article has been updated.

