# Supplementary material for: Clinical outcomes of DMEK comparing endothelium-out injector and endothelium-in pull-through techniques in Asian eyes
Source: Front Med (Lausanne). 2025 Apr 3;12:1555620. doi: 10.3389/fmed.2025.1555620 (PMC12003366; doi:10.3389/fmed.2025.1555620)
Supplement: Supplementary file 1 [file Table_1.docx]

**Supplementary Table 1**: Comparison of visual outcomes, intraoperative, postoperative complications and final graft outcomes between injector and pull-through surgical techniques within the sub-group of FECD eyes.

| **Outcomes** | **All eyes (FECD)** *n* = 103 |  | **Insertion technique** | | ***P*** | |
| --- | --- | --- | --- | --- | --- | --- |
|  |  |  | **Injector** | **Pull-through** |  |  |
|  |  |  | *n* = 92 | *n* = 11 |  |  |
| **Intra-operative complications** | | | | | |  |
| Any complication | 7 (6.8) |  | 6 (6.5) | 1 (9.1) | 0.558 | |
| Donor graft tear | 1 (1.0) |  | 1 (1.1) | 0 | 1.00 | |
| Aqueous misdirection | 1 (1.0) |  | 1 (1.1) | 0 | 1.00 | |
| Hyphaema | 4 (3.9) |  | 3 (3.3) | 1 (9.1) | 0.368 | |
| High vitreous pressure | 0 |  | 0 | 0 | 1.00 | |
| Decentered graft | 1 (1.0) |  | 1 (1.1) | 0 | 1.00 | |
| **Post-operative complications** | |  |  |  |  | |
| Any complication | 16 (15.5) |  | 15 (16.3) | 1 (9.1) | 1.00 | |
| Cystoid macula edema | 4 (3.9) |  | 4 (4.4) | 0 | 1.00 | |
| Early rejection signs | 5 (4.9) |  | 4 (4.4) | 1 (9.1) | 0.438 | |
| Partial detachment | 5 (4.9) |  | 5 (5.4) | 0 | 1.00 | |
| Complete detachment | 1 (1.0) |  | 1 (1.1) | 0 | 1.00 | |
| Rebubbling required | 3 (2.9) |  | 3 (3.3) | 0 | 1.00 | |
| Corneal haze/edema | 4 (3.9) |  | 4 (4.4) | 0 | 1.00 | |
| Ocular hypertension | 4 (3.9) |  | 4 (4.4) | 0 | 1.00 | |
| Retinal detachment | 1 (1.0) |  | 1 (1.1) | 0 | 1.00 | |
| **Final graft outcome** |  |  |  |  |  | |
| Clear & surviving | 101 (98.1) |  | 90 (97.8) | 11 (100) | 1.00 | |
| Graft failure | 2 (1.9) |  | 2 (2.2) | 0 |  |  |
| **Visual outcomes (logMAR)** |  |  |  |  |  | |
| Pre-DMEK BCVA | 0.68 ± 0.42 |  | 0.67 ± 0.40 | 0.81 ± 0.57 | 0.299 | |
| Post-DMEK BCVA* | 0.22 ± 0.31 |  | 0.23 ± 0.32 | 0.14 ± 0.10 | 0.358 | |
| Post-DMEK BCVA ≥ 6/12 | 85 (82.5) |  | 75 (81.5) | 10 (90.9) | 0.684 | |
| % improvement in BCVA | 65.3 ± 38.9 |  | 63.9 ± 40.2 | 76.7 ± 24.0 | 0.307 | |
| logMAR: logarithm of minimum angle of resolution, BCVA: best corrected visual acuity. * Post-op BCVA defined as best BCVA score within 24-months post-operatively. | | | | | | |
