# Supplementary material for: Clinical outcomes of DMEK comparing endothelium-out injector and endothelium-in pull-through techniques in Asian eyes
Source: Front Med (Lausanne). 2025 Apr 3;12:1555620. doi: 10.3389/fmed.2025.1555620 (PMC12003366; doi:10.3389/fmed.2025.1555620)
Supplement: Supplementary file 2 [file Table_2.docx]

**Supplementary Table 2**: Comparison of visual outcomes, intraoperative, postoperative complications and final graft outcomes between injector and pull-through surgical techniques within the sub-group of PBK eyes.

| **Outcomes** | **All eyes (PBK)** *n* = 77 |  | **Insertion technique** | | ***P*** | |
| --- | --- | --- | --- | --- | --- | --- |
|  |  |  | **Injector** | **Pull-through** |  |  |
|  |  |  | *n* = 26 | *n* = 51 |  |  |
| **Intra-operative complications** | | | | | |  |
| Any complication | 14 (18.2) |  | 4 (15.4) | 10 (19.6) | 0.762 | |
| Donor graft tear | 4 (5.1) |  | 0 | 4 (7.8) | 0.294 | |
| Aqueous misdirection | 2 (2.6) |  | 1 (3.9) | 1 (2.0) | 1.00 | |
| Hyphaema | 4 (5.1) |  | 2 (7.7) | 2 (3.9) | 0.600 | |
| High vitreous pressure | 2 (2.6) |  | 1 (3.9) | 1 (2.0) | 1.00 | |
| Decentered graft | 4 (5.1) |  | 1 (3.9) | 3 (5.9) | 1.00 | |
| **Post-operative complications** | |  |  |  |  | |
| Any complication | 32 (41.6) |  | 11 (42.3) | 21 (41.2) | 1.00 | |
| Cystoid macula edema | 3 (3.9) |  | 2 (7.7) | 1 (2.0) | 0.262 | |
| Early rejection signs | 3 (3.9) |  | 1 (3.9) | 2 (3.9) | 1.00 | |
| Partial detachment | 8 (10.4) |  | 4 (15.4) | 4 (7.8) | 0.432 | |
| Complete detachment | 2 (2.6) |  | 0 | 2 (3.9) | 0.547 | |
| Rebubbling required | 5 (6.5) |  | 2 (7.7) | 3 (5.9) | 1.00 | |
| Corneal haze/edema | 16 (20.8) |  | 4 (15.4) | 12 (23.5) | 0.556 | |
| Ocular hypertension | 5 (6.5) |  | 1 (3.9) | 4 (7.8) | 0.657 | |
| Retinal detachment | 1 (1.3) |  | 0 | 1 (2.0) | 1.00 | |
| **Final graft outcome** |  |  |  |  |  | |
| Clear & surviving | 68 (88.3) |  | 23 (88.5) | 45 (88.2) | 1.00 | |
| Graft failure | 9 (11.7) |  | 3 (11.5) | 6 (11.8) |  |  |
| **Visual outcomes (logMAR)** |  |  |  |  |  | |
| Pre-DMEK BCVA | 1.33 ± 0.62 |  | 1.07 ± 0.50 | 1.46 ± 0.64 | **<0.01**** | |
| Post-DMEK BCVA* | 0.40 ± 0.41 |  | 0.27 ± 0.28 | 0.47 ± 0.45 | **0.042*** | |
| Post-DMEK BCVA ≥ 6/12 | 47 (61.0) |  | 19 (73.1) | 28 (54.9) | 0.144 | |
| % improvement in BCVA | 67.9 ± 30.0 |  | 73.4 ± 27.4 | 65.2 ± 31.2 | 0.262 | |
| logMAR: logarithm of minimum angle of resolution, BCVA: best corrected visual acuity. * Post-op BCVA defined as best BCVA score within 24-months post-operatively. | | | | | | |
